# Does palliative care improve the quality of life for cancer patients in three tertiary hospitals in South Africa?

**DOI:** 10.1186/s12904-025-01861-9

**Published:** 2025-08-28

**Authors:** Mpho Ratshikana, Witness Mapanga, Sukoluhle Pilime, Peedi Mathobela, Oluwatosin Ayeni, Anu Abrahams, Lawrence Mandikiana, Phillip Makume, Sonti Imogene Pilusa, Jacob Merika Tsitsi

**Affiliations:** 1 Faculty of Health Sciences, Wits Centre for Palliative Care, University of Witwatersrand, Johannesburg, South Africa; 2 Department of Internal Medicine, Chris Hani Baragwanath Academic Hospital Johannesburg, Johannesburg, South Africa; 3 Faculty of Health Sciences, School of Public Health, University of Witwatersrand, Johannesburg, South Africa; 4 Department of Radiation Sciences, Division of Radiation Oncology, Faculty of Health Sciences, University of the Witwatersrand Johannesburg, Johannesburg, South Africa; 5 Faculty of Health Sciences, Strengthening Oncology Services Research Unit, University of the Witwatersrand, Johannesburg, South Africa; 6 Department of Internal Medicine, Charlotte Maxeke Johannesburg Academic Hospital, Johannesburg, South Africa; 7 Physiotherapy Department, Faculty of Health Sciences, University of the Witwatersrand, Johannesburg, South Africa; 8 Department of Internal Medicine, Faculty of Health Sciences, University of Witwatersrand, Johannesburg, South Africa; 9 Faculty of Health, School of Public Health Sciences, University of Waterloo, Waterloo, Canada

**Keywords:** Cancer, Quality of life, Palliative care, Comprehensive, Cancer care

## Abstract

**Background:**

Quality of life (QoL) is a crucial treatment outcome for cancer patients, who often experience significant symptom burden and distress. Despite its benefits, access to palliative care remains limited in many settings, including South Africa. This study assessed changes in QoL before and after palliative care interventions and examined factors associated with QoL improvements.

**Methods:**

A retrospective cohort study was conducted among cancer patients referred for palliative care at three tertiary hospitals in Johannesburg, South Africa. Interdisciplinary teams provided palliative care, and patient data on sociodemographic and clinical characteristics were collected. QoL was measured using the World Health Organization Quality Of Life-BREF (WHOQOL BREF) tool at baseline and follow-up. Paired t-tests compared mean QoL scores, and mixed regression analysis identified factors associated with QoL improvements. Data were analyzed using Stata version 18 SE.

**Results:**

Among 724 patients (68.51% female, mean age 51.78 years, SD: 16.62), the most common cancers were breast (29.83%), gastrointestinal/hepatobiliary (21.27%), and cervical (16.71%). Baseline QoL mean (SD) scores were low across all domains. Following palliative care, significant improvements (*p* < 0.001) were observed: general health improved from 43.64 (26.17) to 66.28 (26.71); physical health from 48.46 (15.45) to 53.42 (15.08); psychological health from 57.54 (17.39) to 65.44 (18.75); environmental health from 60.97 (19.90) to 71.49 (18.84) and social health from 59.26 (25.60) to 71.08 (23.54) (all *p* < 0.001). HIV-negative status was associated with better outcomes across all domains, with coefficients ranging from 0.19 to 0.46 (*p* < 0.05) compared to those living with HIV.

**Conclusions:**

Palliative care significantly improved QoL across all measured domains among cancer patients at tertiary hospitals in Johannesburg. These findings highlight the need to integrate palliative care into routine oncology treatment to enhance patient well-being.

## Background

Cancer is a global public health challenge that not only affects the physical health of individuals but also influences their overall quality of life (QOL) [1]. Globally, over 10 million people die from cancer annually, 70% of whom are from low- and middle-income countries (LMICs) [2]. An estimated 19.3 million new cancer cases were reported in 2020, and 1.1 million of these were from Africa [3]. In sub-Saharan Africa (SSA), cancer incidence and mortality have increased in the past 30 years and are predicted to double between 2018 and 2040 [4, 5]. According to the South African Cancer Registry report, in 2020, 76,427 new cancer cases were reported [6], while cancer deaths increased by 60% between 1997 and 2018 [7].

The World Health Organization (WHO) defines QOL as an “individual’s perceptions of their position in life in the context of their culture and value systems in which they live and concerning their goals, expectations, standards and concerns” [8]. Self-reported QOL is an important measure of patient treatment outcomes and is recommended as part of cancer care [9]. Several studies report low quality of life among cancer patients from LMICs [10–14]. In a systematic review of 27 articles, Qanir et al. found that the overall QOL among cancer patients in SSA is low and is associated with socio-demographic and clinical characteristics [15]. Routine measurement and tracking of QOL is crucial among cancer patients to reduce hospitalisation and improve treatment outcomes and survival [16].

Several factors influence QOL in cancer patients. Among these are co-morbidities, level of education [17, 18], employment status [17], clinical staging [17], symptom burden [1, 17, 19], and psychosocial and spiritual distress [1, 20, 21]. Cancer patients have a high burden of symptoms, which has been found to negatively affect their QoL [13]. Some of the symptoms include pain, drowsiness, fatigue, dry mouth, and dyspnoea [20, 21]. Addressing the physical, psychosocial and spiritual challenges cancer patients face through palliative care is associated with improved quality of life [22].

Palliative care is an approach that aims to relieve suffering and improve the QoL of patients, and is recommended from diagnosis of cancer till the bereavement period, providing support for existing family members in the unfortunate event of the patient dying [22]. Through palliative care consultations, patients are provided with clinical, psychosocial, and spiritual support, which has been found to improve their quality of life and treatment outcomes [23]. Though research on the QoL among cancer patients is increasing globally, evidence in South Africa is still limited [23], and there is a lack of data on palliative care access for people diagnosed with cancer, and at what stage they are referred for palliative care. Through this study, we aimed to assess the QoL of cancer patients following palliative care intervention. Our objectives were to: (1) describe the demographic and clinical characteristics of cancer patients receiving palliative care services (2), compare the QoL of cancer patients at baseline and first follow-up after a palliative care intervention, and (3) assess the association between QoL and sociodemographic and clinical characteristics. We postulate that the QoL among cancer patients is low, will improve following palliative care interventions and will be associated with some socio-demographic and clinical characteristics.

## Methods

### Setting and population

The study was conducted at three tertiary public hospitals; Chris Hani Baragwanath Academic Hospital (CHBAH), Charlotte Maxeke Johannesburg Academic Hospital (CMJAH), and Helen Joseph Hospital (HJH). Palliative care services are offered at all three institutions through support from the Wits Centre for Palliative Care. Chris Hani Baragwanath Academic Hospital is the 3rd largest hospital globally and has been providing palliative care for over 20 years. The hospital provides chemotherapy cancer care for adult patients and refers patients who require radiotherapy to CMJAH. CMJAH is the main oncology hospital in Johannesburg, offering both chemotherapy and radiation therapy, and Helen Joseph Hospital also provides tertiary services in the province. All three hospitals are affiliated with the University of Witwatersrand School of Clinical Medicine and provide care to cancer patients mainly from and around Johannesburg, Ekurhuleni, and neighbouring provinces. Patients diagnosed with cancer are referred to palliative care teams in the three hospitals. The teams comprise of doctors, nurses, social workers and social auxiliary workers and spiritual counsellor (chaplains). All team members are trained to provide palliative care for referred patients.

### Study design and intervention

This was a retrospective cohort study of cancer patients referred for palliative care services at CHBAH, CMJAH, and HJH between June 2021 to January 2023. Included in the study were cancer patients older than 18 years, confirmed with a confirmed histological cancer diagnosis, and able to respond to questions during palliative care consultations. During the first palliative care consultation, trained staff (nurses, doctors, social workers and spiritual counsellors) routinely collected baseline information, assessed palliative care needs and provided palliative care interventions. Information was collected on the socio-demographic factors i.e., (age, gender, marital status, education level, employment status), and clinical characteristics (histology, clinical staging, weight loss, HIV status, and other comorbidities) from the patients. Data on functional status was collected using the Eastern Cooperative Oncology Group Performance Status Tool (ECOG-PS) [24]. The ECOG PS tool is a globally validated tool that is used to measure the performance status of patients. The tool has five gradings ranging from zero to five; ECOG 0 indicates patients who are able to carry out daily activities without restriction; ECOG 1 indicates ambulant patients who have some restricted physical activity but able carry out light duties; ECOG 2 refers to ambulant patients only capable of self-care, but up for more than 50% of the waking hours, ECOG 3 patients are have limited self-care, and in bed for more than 50% of the day; ECOG 4 are patients who are completely bedridden and incapable of self-care, while ECOG 5 denoted death.

Baseline QoL was assessed using the World Health Organization Quality Of Life-BREF (WHOQOL BREF) [25]. The WHOQOL BREF questionnaire has six domain scores, 24 facet scores, and one general facet score that measures overall QoL and general health. The five domains include physical (3 questions), psychological (5 questions), social (3 questions) and environmental (8 questions) [25]. The scores are scaled in a positive direction ranging from 1 to 5 i.e. higher scores denote higher QoL. However, some facets (pain and discomfort, negative feelings, dependence on medication) are not scaled in a positive direction. This means that for these facets higher scores (e.g. 5) denote lower quality of life, and lower scores (e.g. 1) denote higher quality of life. Therefore, they were reverse scored to be scaled in the positive direction. The five domain scores denote an individual’s perception of QoL in each domain. The mean scores of items within each domain are used to calculate the domain score. The mean scores are then multiplied by 4 in order to make the domain scores comparable with the scores used in the WHOQOL-100, and then transformed to a 0-100 scale. Explicit instructions for checking and cleaning the data, and for computing the domain scores are given in page 106 (Appendix 10) of the WHOQOL user manual [8].

After an initial assessment of baseline QoL, referred patients were managed by the interdisciplinary palliative care team comprising doctors, nurses, social workers, and spiritual counsellors. The patients received comprehensive palliative care assessment on their physical, social, emotional and spiritual needs., and based on the needs identified, clinicians managed the physical symptoms, social workers addressed psychological challenges and spiritual counsellors provided spiritual care. Palliative care interventions were provided in the hospital wards, hospital outpatient clinics, and in patients’ homes. As part of routine, repeat WHOQOL-BREF is re-administered on patients within a period of two to four weeks after the initial palliative care intervention to assess progress on their quality of life, after which the WHOQOL-BREF tool is repeated monthly. We analysed QoL data which were collected from the first consultation (baseline) and the first follow-up visit and compared the scores to assess the effect of palliative care intervention. We carried out a sensitivity analysis to compare the QoL of the 724 participants with baseline and follow-up quality assessment data with 2,135 participants with imputed data i.e. those with baseline QoL and no follow-up QoL. The results showed that the mean QoL domain scores were not different between participants with baseline and follow up QoL and those with imputed data.

### Statistical analysis

**Fig. 1:**
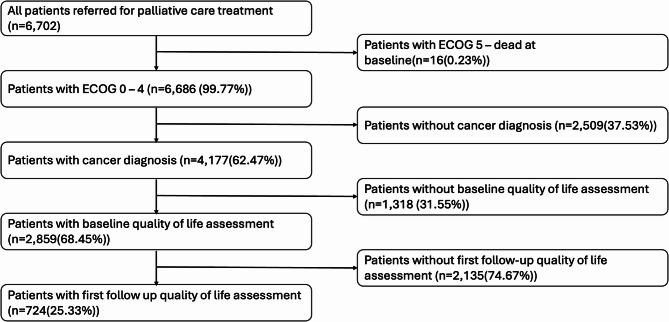
Flow diagram showing palliative care patients from June 2021 to January 2023 that were included in the study

Data were extracted from the REDCap [24, 25] database maintained at the Gauteng Centre for Palliative Care at Chris Hani Baragwanath Academic Hospital. A flow diagram of the palliative care patients which were included in the study is shown in Fig. 1. We described the sociodemographic and clinical characteristics of the patients diagnosed with cancer using means ± standard deviation (SD) for continuous variables and frequencies with percentages for categorical variables.

We compared the baseline QoL with the QoL at the 2nd palliative care assessment (first follow-up) of the patients. This was done using paired t-tests to determine the differences in means between baseline and first follow-up quality of life scores after a psychological and spiritual intervention. Means ± SD were reported for each question, and domain. A 2-sided p-value of less than 0.05 was considered significant throughout. Analysis was done using Stata version 18 SE (Stata Corp LP, College Station, TX). Mixed regression was used to analyse factors associated with each QOL domain.

## Results

### Demographic characteristics of patients

A total of 724 cancer patients with complete data on baseline and follow-up quality-of-life assessments were included. The mean age of participants was 51.78 (SD-14.62). Most patients were black (81.08%), female (68.51%), and 41.8% were married or in relationships. Out of the patients who responded on employment status, 52,93% were unemployed. Most of the patients had a high and technical school qualification (76,38%), and only 3,31% had no formal education. Table 1 shows the demographic characteristics of cancer patients who received palliative care at the three hospitals.

**Table 1:** Demographic characteristics of patients with a cancer diagnosis who received who received palliative care at the three study sites

Covariate	Mean (SD)
Age	51.78 (14.62)
Factors	Frequency (%)
*Sex*	*N=724*
Male	228 (31.49%)
Female	496 (68.51%)
*Race*	*N=724*
Black	587 (81.08%)
White	75 (10.36%)
Mixed race (Coloured)	45 (6.22%)
Other	17 (2.35%)
*Marital status*	*N=720*
Married/partnered	301 (41.81%)
Single	418 (58.06%)
Refused to say	1 (0.14%)
*Employment status*	*N=682*
Employed	163 (23.90%)
Unemployed	361 (52.93%)
Retired	158 (23.17%)
*Level of education*	*N=635*
No formal education	21 (3.31%)
Primary school	43 (6.77%)
High and technical school	485 (76.38%)
Graduate-Tertiary education	86 (13.54%)

### Clinical characteristics of patients

As shown in Table 2, the five most common cancers were breast (29.83%) and gastrointestinal, including hepatobiliary (21.27%), cervix (16.71%), lung (8.80%), and prostate (5.25%). A significant proportion of cancer patients presented late, clinical stage III (26.21%) and stage IV (27.88%), while 280 patients (39.05%) were HIV positive, and 29.90% had other chronic diseases (e.g., heart disease, diabetes etc.,). Regarding performance and functional status, over one third of the patients had functional impairment (ECOG 3; 30.25% and ECOG 4; 7.18%).

**Table 2:** Clinical characteristics of patients with a cancer diagnosis who received palliative care at the three study sites

Factors	Frequency (%)
Type of cancer	*N* = 724
Breast	216 (29.83%)
Cervix	121 (16.71%)
GIT/Hepatobiliary	154 (21.27%)
Lung	64 (8.84%)
Prostate	38 (5.25%)
Other	131 (18.09%)
*Clinical stage*	*N* = 721
Stage 1	22 (3.05%)
Stage 2	125 (17.34%)
Stage 3	189 (26.21%)
Stage 4	201 (27.88%)
Unknown	184 (25.52%)
*ECOG*	*N* = 724
0	46 (6.35%)
1	168 (23.21%)
2	239 (33.01%)
3	219 (30.25%)
4	52 (7.18%)
*HIV status*	*N* = 717
Positive	280 (39.05%)
Negative	437 (60.95%)
*TB*	*N* = 619
Positive	10 (1.62%)
Negative	609 (98.38%)
*Chronic disease*	*N* = 719
Yes	215 (29.90%)
No	504 (70.10%)

### Time taken from baseline assessment to first follow-up session

For this study, the median (IQR) time for patients to be assessed at first follow-up was 14 days (6–51 days). Approximately 65% of the patients enrolled for palliative care had their first follow-up quality of life assessment within a month.

### Quality of life scores at baseline and first follow-up

Overall, our results showed a statistically significant improvement in the QoL of cancer patients following palliative care intervention as shown in Table 3. Patients reported an increase in their enjoyment of life, an improvement in their perception of the meaningfulness of life, and improved energy. The study results showed that additional questions on the QoL had the same trend, where palliative care interventions positively improved the QoL of cancer patients. This included the ability to concentrate, feelings of safety, and the ability to sleep well. Of note, the mean for medication needed to function in daily life decreased at the follow-up visit. Physical pain, which affects activities of daily living, improved and negative feelings were less burdensome for the participants.

**Table 3:** Comparison of mean ± sd quality of life (WHOQOL-BREF) scale items scores between baseline and first follow-up visits for cancer patients who received palliative care at the three study sites

Questions	Baseline mean ± sd	1 st follow-up mean ± sd	*p*-value (CI)
How would you rate your quality of life?	2.96 ± 1.07	3.87 ± 1.03	*P* < 0.001 [−0.96; −0.81]
How satisfied are you with your health?	2.52 ± 1.20	3.43 ± 1.24	*P* < 0.001 [−0.99; −0.80]
To what extent do you feel that (physical) pain prevents you from doing what you need to do?	2.87 ± 1.26	2.35 ± 1.18	*P* < 0.001 [0.40; 0.60]
How much do you need any medical treatment to function in your daily life?	3.68 ± 1.12	3.40 ± 1.04	*P* < 0.001 [0.19; 0.36
How much do you enjoy life?	3.24 ± 1.20	3.78 ± 1.23	*P* < 0.001 [−0.63; −0.45]
To what extent do you have the opportunity for leisure activities?	2.60 ± 0.04	2.98 ± 0.04	*P* < 0.001 [−0.32; −0.18]
How well are you able to concentrate?	3.50 ± 0.93	3.84 ± 0.95	*P* < 0.001 [−0.39; −0.25]
How safe do you feel in your daily life?	3.63 ± 1.00	4.12 ± 0.90	*P* < 0.001 [−0.55; −0.39]
How healthy is your physical environment?	3.93 ± 0.95	4.28 ± 0.86	*P* < 0.001 [−0.39; −0.24]
Do you have enough energy for everyday life?	2.90 ± 1.06	3.27 ± 1.13	*P* < 0.001 [−0.43; −0.27]
Are you able to accept your bodily appearance?	3.17 ± 1.30	3.73 ± 1.33	*P* < 0.001 [−0.62; −0.42]
Have you enough money to meet your needs?	2.59 ± 1.08	2.94 ± 0.99	*P* < 0.001 [−0.52; −0.38]
How available to you is the information that you need in your day-to-day life?	3.77 ± 0.93	4.26 ± 0.85	*P* < 0.001 [−0.47; −0.30]
How well are you able to get around?	3.16 ± 1.10	3.42 ± 1.14	*P* < 0.001 [−0.32; −0.17]
How satisfied are you with your sleep?	3.09 ± 1.14	3.85 ± 1.10	*P* < 0.001 [−0.79; −0.60]
How satisfied are you with your ability to perform your daily living activities?	2.67 ± 1.23	3.16 ± 1.31	*P* < 0.001 [−0.57; −0.39]
How satisfied are you with your work capacity?	2.14 ± 1.20	2.45 ± 1.26	*P* < 0.001 [−0.41; −0.24]
How satisfied are you with yourself?	2.92 ± 1.27	3.76 ± 1.24	*P* < 0.001 [−0.88; −0.69]
How satisfied are you with your relationships?	3.58 ± 1.21	4.15 ± 1.01	*P* < 0.001 [−0.60; −0.43]
How satisfied are you with your sex life?	2.83 ± 1.29	3.27 ± 1.32	*P* < 0.001 [−0.52; −0.34]
How satisfied are you with the support you get from your friends?	3.63 ± 1.05	4.04 ± 0.95	*P* < 0.001 [−0.45; −0.29]
How satisfied are you with the conditions of your living place?	3.74 ± 1.07	4.19 ± 0.90	*P* < 0.001 [−0.49; −0.33]
How satisfied are you with your access to health services?	3.75 ± 0.98	4.24 ± 0.89	*P* < 0.001 [−0.53; −0.37]
How satisfied are you with your transport?	3.43 ± 1.07	3.78 ± 1.07	*P* < 0.001 [−0.39; −0.22]
How often do you have negative feelings such as blue mood, despair, anxiety, and depression?	2.72 ± 1.02	2.09 ± 0.80	*P* < 0.001 [0. 53; 0. 69]

### Assessment of quality of life by domains

There was significant improvement in quality-of-life scores for all domains (*p* < 0.001) at first follow-up after a palliative care intervention (Table 4): general health from 43.64 (26.17) to 66.28 (26.71); physical health from 48.46 (15.45) to 53.42 (15.08); psychological health from 57.54 (17.39) to 65.44 (18.75); environmental health from 60.97 (19.90) to 71.49 (18.84) and social health from 59.26 (25.60) to 71.08 (23.54).

**Table 4:** Mean scores for WHOQOL-BREF domains measured at baseline and first quality of life follow-up assessment

Domains	Domain score range	Baseline: mean (SD)	1 st follow-up: mean (SD)	*p*-value (CI)
General Health	0-100	43.09 (25.80)	65.30 (26.38)	*P* < 0.001 [−24.11; −20.30]
Physical health	0-100	47.98 (15.36)	52.66 (15.06)	*P* < 0.001 [−5.79; −3.57]
Psychological health	0-100	56.98 (17.21)	64.42 (18.71)	*P* < 0.001 [−8.71; −6.18]
Environmental Health	0-100	60.45 (19.84)	70.21 (19.09)	*P* < 0.001 [−11.19; −8.32]
Social health	0-100	58.75 (25.30)	69.69 (23.55)	*P* < 0.001 [−12.67; −9.22]

Table 5 indicates the mixed effects regression analysis on factors associated with physical, psychological, environmental, and social health QOL domains. There were significant improvements from baseline to follow-up across all QOL domains (p = < 0.0001); physical: 2.67e-15 [95%CI: 2.31e-15: 3.03e-15]; psychological: 7.09e-15 [95%CI: 6.47e-15: 7.70e-15]; environmental: 4.47e-15 [95%CI: 3.92e-15: 5.01e-15]; and social: 5.88e-15 [95%CI: 5.27e-15: 6.50e-15] health domains. The psychological health domain scores were significantly higher in Cervical cancer patients compared to Breast cancer patients. When compared to Breast cancer patients, the physical, psychological, environmental, and social health domains in Lung cancer patients were significantly lower.

**Table 5:** Mixed effects regression analysis on factors associated with physical health, psychological, level of independence, environmental and social health QoL domains

QOL Domains	Physical health: β(95% CI) (*p*-value)	Psychological health: β(95% CI) (*p*-value)	Environmental health: β(95% CI) (*p*-value)	Social health: β(95% CI) (*p*-value)
Contact Period				
Baseline	Reference			
Follow up	2.67e-15 (2.31e-15: 3.03e-15) (< 0.0001)**	7.09e-15 (6.47e-15: 7.70e-15) (< 0.0001)**	4.47e-15 (3.92e-15: 5.01e-15) (< 0.0001)**	5.88e-15 [5.27e-15; 6.50e-15] (< 0.0001)**
Cancer type				
Breast	Reference			
Cervix	1.63 (−1.34: 4.60) (0.281)	5.13 (1.63: 8.62) (0.004)**	3.70 (−0.42: 7.82) (0.078)	4.41 [−0.46; 9.28] (0.076)
GIT/Hepatobiliary	−0.29 (−3.13: 2.54) (0.839)	−0.17 (−3.89, 3.54) (0.928)	0.84 (−2.99: 4.67) (0.667)	0.68 [−4.08; 5.44] (0.780)
Lung	−5.42 (−8.93: −1.91) (0.002)**	−10.47 (−15.26: −5.67) (< 0.001)**	−5.47 (−10.44: −0.51) (0.031)**	−8.10 [−14.21; −1.99] (0.009)**
Prostate	1.65 (−2.33: 5.64) (0.416)	−0.27 (−6.65: 6.11) (0.993)	−1.18 (−7.90: 5.55) (0.731)	−4.36 [−12.62; 3.89] (0.300)
Other	0.57 (−2.29: 3.43) (0.695)	4.23 (0.61: 7.85) (0.022)**	2.70 (−1.17: 6.56) (0.172)	4.59 [−0.11; 9.29] (0.056)
ECOG				
0	Reference			
1	−5.28 (−9.58, −0.98) (0.016)**	−4.97 (−9.31: −0.62) (0.025)	−4.70 (−10.49: 1.09) (0.111)	−5.51 [−13.25; 2.23] (0.163)
2	−8.51 (−12.32: −4.36) (< 0.0001)**	−6.58 (−10.75: −2.41) (0.002)**	−4.93 (−10.57: −0.71) (0.087)	−5.74 [−13.38; 1.89] (0.140)
3	−18.16 (−22.31: −14.00) (< 0.0001)**	−15.00 (−19.19: −10.82) (< 0.0001)**	−13.64 (−19.28: −8.00) (< 0.0001)**	−16.47 [−24.10; −8.84] (< 0.0001)**
4	−22.97 (−28.43, −17.51) (< 0.0001)**	−22.25 (−28.79: −15.71) (< 0.0001)**	−21.23 (−28.59: −13.87) (< 0.0001)**	−21.85 [−31.20; −12.50] (< 0.0001)**
HIV status				
Positive	Reference			
Negative	4.40 (2.42: 6.39) (< 0.0001)**	7.20 (4.68: 9.73) (< 0.0001)**	5.93 (3.16: 8.70) (< 0.0001)**	10.84 [7.48; 14.20] (< 0.0001)**
Employment status				
Employed	Reference			
Unemployed	1.66 (−0.58: 3.89) (0.146)	2.85 (−0.04: 5.74) (0.053)	0.24 (−2.82: 3.31) (0.877)	3.13 [−0.67: 6.94] (0.107)
Retired	1.03 (−1.70: 3.77) (0.459)	3.57 (0.07: 7.07) (0.045)**	4.98 (1.39: 8.57) (0.007)**	5.28 [0.80; 9.76] (0.021)**
Level of education				
No formal education			Reference	
Primary school	-	-	−1.90 (−11.55: 7.74) (0.699)	-
High and technical school	-	-	0.70 (−7.56: 8.95) (0.869)	-
Graduate-Tertiary education	-	-	2.40 (−6.40: 11.20) (0.593)	-

Key; (β) represents coefficient, (-) means the variable was not included in the final model because it was not significant, (**) means *p*-value<0.05

Higher ECOG performance status was consistently linked to declines across QoL outcomes. Patients with 1, 2, 3, and 4 ECOG levels (lower functional status) had significantly lower physical and psychological health QoL than patients with an ECOG status of zero. Similarly, patients with ECOG 3, and 4 had significantly lower environmental and social health QoL than patients with an ECOG of zero.

Patients with a negative HIV status were associated with better outcomes across all domains compared those living with HIV. The effect of employment status varied; unemployment had a limited positive effect, while retirement positively influenced the psychological, environmental, and social domains.

## Discussion

This study explored the role of palliative care interventions on the QoL of cancer patients treated at three tertiary hospitals in Johannesburg, South Africa. The findings highlight a significant improvement in patients’ QoL across multiple domains, including physical, psychological, social, and environmental health. The observed improvements in QoL align with the findings from other studies in low- and middle-income countries (LMICs) that have emphasised the critical role of palliative care in enhancing cancer patients’ QoL [11, 14].

The baseline assessments indicated notably low QoL scores in general and physical health domains, with averages of 43.64 and 48.46, respectively lower compared to those reported in high-income settings [26, 27]. This aligns with prior studies that underscore the high symptom burden and psychological distress experienced by cancer patients in resource-limited settings [10, 13]. This disparity underscores the ongoing challenges in palliative care delivery in resource-constrained environments, including limited access to specialised services and medications [28, 29].

Following the initial palliative care interventions, there were statistically significant improvements in all QoL domains. For instance, general health scores increased from 43.64 to 66.28 (*p* < 0.001), while physical health improved from 48.46 to 53.42 (*p* < 0.001). These findings affirm the positive role of palliative care in alleviating physical symptoms such as “pain” and enhancing patients’ perception of overall well-being [29–31]. Physical pain, a common debilitating symptom among cancer patients, significantly reduced following palliative care. The mean score for the extent to which pain limited daily activities decreased from 2.87 to 2.35 (*p* < 0.001). This improvement demonstrates the effectiveness of pain management strategies in palliative care, consistent with findings by Harding et al. [32].

The psychological domain exhibited marked improvement, with scores increasing from 57.54 at baseline to 65.44 at the first follow-up (*p* < 0.001). This finding highlights the significance of addressing psychological distress through palliative interventions, as previously reported by Grassi et al. [33]. Enhanced social support and spiritual counselling likely contributed to this positive outcome. Patients also reported feeling safer, enjoying life more, and having fewer negative feelings post-intervention, further supporting evidence that comprehensive care strategies improve psychological outcomes [29, 34, 35]. This study underscores the need for routine integration of palliative care into oncology treatment pathways, starting from the point of diagnosis. Early palliative care interventions not only alleviate symptom burden but also enhance patients’ psychological resilience and social support systems [23, 30]. This finding is particularly relevant in South Africa, where cancer patients often present at advanced stages of disease [36].

Furthermore, regression analysis revealed that certain factors were significantly associated with QoL improvements. For example, patients with higher Eastern Cooperative Oncology Group (ECOG) performance statuses (i.e., more functional impairment) showed smaller gains in QoL, highlighting the importance of early palliative care referral [37]. HIV-negative patients also exhibited greater improvements in QoL across domains, reflecting the compounded health challenges faced by HIV-positive individuals [38]. Efforts should be made to address factors associated with QoL improvement and other barriers to palliative care access, including workforce shortages, stigma, and inadequate training of healthcare provider [31]. Furthermore, palliative care should be integrated into HIV care, and policymakers should consider investing in the expansion of home-based care services and ensuring equitable access to essential medications for pain management.

### Limitations and future research

The study’s retrospective design and reliance on self-reported QoL assessments are potential limitations, as recall bias may have influenced the results. The study did not consider other treatment modalities, like chemotherapy, radiation therapy and treatment provided by primary carers. Additionally, the follow-up period of approximately two weeks may have been insufficient to capture long-term outcomes of the interventions. Future studies should consider longer follow-up periods and explore association between specific palliative care interventions (pharmacological, emotional, social and spiritual) and oncology treatment with the QoL.

## Conclusions

This study provides compelling evidence that palliative care interventions significantly improve the QoL of cancer patients in Johannesburg. The findings support the routine incorporation of palliative care into comprehensive cancer management strategies. Further research is needed to optimise the delivery of palliative care services and ensure their accessibility to all patients in need.

## Data Availability

Data that were analyzed for this study is available upon request. It is securely stored in the Wits Centre for palliative Care database, managed under the Chris Hani Baragwanath Academic Hospital Department of Internal Medicine, in the Palliative Care Department.

## Abbreviations

CHBAH: Chris Hani Baragwanath Academic Hospital
CMJAH: Charlotte Maxeke Johannesburg Academic Hospital
ECOG: Eastern Cooperative Oncology Group
ECOG-PS: Eastern Cooperative Oncology Group Performance Status Tool
HJH: Helen Joseph Hospital
LMIC: Low- and middle-income countries
QoL: Quality of life
SD: Standard deviation
SSA: Sub-Saharan Africa
WHO: World Health Organization
